# Low mean HbA1c does not increase all-cause and cardiovascular mortality in patients with diabetes: Effect-modifications by anemia and chronic kidney disease stages

**DOI:** 10.1371/journal.pone.0272137

**Published:** 2022-08-11

**Authors:** Seng-Wei Ooi, Shu-Tin Yeh, Ya-Hui Chang, Chung-Yi Li, Hua-Fen Chen

**Affiliations:** 1 Department of Endocrinology, Far Eastern Memorial Hospital, New Taipei City, Taiwan; 2 Department of Public Health, College of Medicine, National Cheng Kung University, Tainan, Taiwan; 3 Department of Public Health, College of Public Health, China Medical University, Taichung, Taiwan; 4 Department of Healthcare Administration, College of Medical and Health Science, Asia University, Taichung, Taiwan; 5 School of Medicine and Department of Public Health, College of Medicine, Fujen Catholic University, New Taipei City, Taiwan; Faculty of Medicine, Saint-Joseph University, LEBANON

## Abstract

**Background:**

Previous studies reported that low levels of glycated hemoglobin A1c (HbA1c) were associated with increased mortality. We investigated rates and risks of all-cause and cardiovascular mortality in association with mean HbA1c levels with stratification of anemia and chronic kidney disease (CKD) stages, major causes of low HbA1c.

**Methods:**

47,145 patients with prescription of antidiabetic agents >6 months in the outpatient visits (2003–2018) were linked to Taiwan’s National Death Registry to identify all-cause and cardiovascular mortality. Poisson assumption was used to estimate the mortality rates, and the Cox proportional hazard regression model was used to evaluate the relative hazards of respective mortality in relation to mean HbA1c in different statuses of anemia and CKD stages.

**Results:**

All-cause and cardiovascular mortality rates were the lowest in non-anemic stages 1–2 CKD patients, and the highest in anemic stages 3–5 CKD patients. In stages 1–2 CKD, excessive HRs observed in those with mean HbA1c <6.0% (Hazard Ratio [HR]) 1.58; 95% Confidence Interval [CI] 1.18–2.12) became inconsequential after adjustment of medications and laboratory results (HR: 1.26; 95% CI 0.89–1.79). The similar patterns were observed in anemic stages 1–2 CKD, anemic or non-anemic stages 3–5 CKD. Low HbA1c was not related to cardiovascular mortality in any anemia status or CKD staging.

**Conclusions:**

Higher risks associated with low mean HbA1c and all-cause mortality were attenuated by adjustment of medications and comorbidities. It is imperative for the diabetologists to consider confounding effects of underlying illness before concluding low HbA1c associated higher mortality.

## Introduction

Glycated hemoglobin A1c (HbA1c) measurement, to assess average glycemia over approximately 3 months [1], is proved to be a strong predictive value for diabetes complications [2,3]. However, some of the previous studies reported that there was a U- [4–6] or J-shaped [7–9] association of mean HbA1c with all-cause mortality; i.e. both low and high levels of HbA1c were associated with increased mortality. One of the above studies analyzed the association of baseline HbA1c and cardiovascular mortality with similar J-shaped [9] association.

As with other laboratory measurements, the HbA1c test is subject to limitations. HbA1c is significantly lower in diabetic patients with anemia compared to the non-anemic diabetic patients [10,11]. Reduced red blood cells (RBC) survival, common in chronic kidney disease (CKD) even in early stages by impairment of erythropoietin production [12], may also affect HbA1c values. Anemia and CKD might have underestimated the measurement of HbA1c, and low HbA1c value detected were likely to be the consequence of underlying illnesses rather than the patient’s true mean glycemia, which subsequently could have confounded the findings from these past analyses. Anemia [13] or CKD [14] itself is reported to be related to higher hazards of all-cause mortality, and can give rise to misinterpretation of low HbA1c-related increased mortality. However, some clinicians assume that the risk of hypoglycemia is highest among patients with lowest HbA1c levels [15], and it would be related to increased mortality [16].

One meta-analysis that was analyzed non-diabetic older adults’ data from cohort studies found out that the significance of J-shaped association of HbA1c with all-cause and cardiovascular mortality lost after adjustment of various confounders including iron-deficiency anemia [17]. However, a recent US National Health and Nutritional Examination Survey (NHANES) 1999–2015 pointed out that after adjustment for various biomarkers including anemia and albumin, low HbA1c was associated with an increased risk of all-cause but not cardiovascular mortality compared with mid-level HbA1c [18].

Although some of the above studies analyzed creatinine [4], estimated glomerular filtration rate (eGFR) [7,8] or prior hospitalization for nephropathy [6] in their models, they did not further estimate the risks of all-cause or cardiovascular mortality in association with different HbA1c in various CKD staging. None of the above studies collected hemoglobin (Hb) levels, and adjusted it in their statistical models.

The aim of our study was to assess the relationship between various levels of mean HbA1c and all-cause and cardiovascular mortality in patients with diabetes treated in Far Eastern Memorial Hospital (FEMH) from Jan 1, 2003 to Dec 31, 2018. Our hypothesis was increased mortality observed in low HbA1c might have been confounded by anemia and CKD. We, therefore, estimated the risks of all-cause and cardiovascular mortality in different levels of anemia and CKD staging with adjustment of medications, comorbidities, and various laboratory results including eGFR and Hb.

## Materials and methods

### Study design and subjects

This was a cohort study designed to evaluate the all-cause and cardiovascular mortality of patients with diabetes in relation to various levels of mean HbA1c with different mean Hb values, CKD staging and prior history of hypoglycemia at FEMH, a tertiary medical center in Northern Taiwan. According to the information from the Taiwan’s National Health Insurance Quality Indicator, the number of outpatient patients with diabetes in FEMH at the end of 2018 was the fourth largest number among 19 medical centers in Taiwan [19]. Twelve endocrinologists take care of nearly 50% of all outpatient patients with diabetes while the rest of the patients are attended by various specialists, mostly by cardiologists, neurologists, nephrologists, and family medicine physicians.

FEMH has established its electronic medical database of out-patient visits since Jan. 1, 2001. These electronic data include information about each patient’s age, sex, hospital chart number, personal identification number (PIN), dates of admission and discharge, length of hospital stay, up to six ICD diagnosis codes, prescribed medications, and laboratory reports including point-of-care (POC) glucometer data. This study was approved by the Research Ethics Review Committee of FEMH (11033-F) with no informed consent required.

From FEMH out-patient database among patients with and without diabetes, we identified 74,888 diabetic subjects with ICD-9-CM: 250.xx or ICD-10-CM: E10 or E11 during the study period between Jan 1, 2003 to Dec 31, 2018. We excluded 4,213 patients without any prescription of oral or parenteral antidiabetic agents, and 23,530 patients with a duration of total outpatient visit less than 6 months at FEMH. The final diabetes cohort consisted of 47,145 patients. The index date of each patient was the first date of oral or parenteral antidiabetic agents prescribed in FEMH during 2003–2018.

### Follow-up, covariates, and study endpoints

The patients were followed-up from the index date to the occurrence of all-cause or cardiovascular mortality or the time of censoring. If the patients did not encounter death during the study period and his/ her HbA1c value was detected beyond Dec 31, 2018, they were censored at the end of the study (i.e. Dec 31, 2018). For other patients, the dates of last out-patient visit in FEMH were set to be their censored dates.

The age of each study subject was set as the difference between the index date and the date of birth. We collected the information of antidiabetic (sulphonylurea, meglitinide, metformin, thiazolidinediones, alpha-glucosidase inhibitors, dipeptidyl peptidase-4 inhibitor, sodium-glucose cotransporter-2 inhibitors, insulin, and glucagon-like peptide-1 receptor agonist), antihypertensive (angiotensin converting enzyme inhibitors, angiotensin receptor blockers, beta-blockers, calcium-channel blockers, and diuretics), and antilipid medications (statin and fibrates) (S1 Table) between the index date and the end of follow-up, which is either death or censoring. Various cardiovascular comorbidities and diabetic complications (coronary artery disease, heart failure, hypertensive disease, cerebrovascular disease, peripheral artery disease, diabetic nephropathy, diabetic retinopathy, and diabetic neuropathy) retrieved from the out-patient medical records between the index date and the end of follow-up (S2 Table) [20] were considered as potential confounders.

The information of each patient’s HbA1c, fasting plasma glucose (FPG), low density lipoprotein (LDL), creatinine (Cr), albumin and Hb level were collected during the study period. HbA1c was measured by ion-exchange high performance liquid chromatography using the automated analysers (G7 and G8, Tosoh Bioscience, Japan [2003–2016] and HA-8180V, Arkray, USA [2017–2018] according to the manufacturers’ instructions. The measurement of HbA1c assay in FEMH is certified by the National Glycohemoglobin Standardization Program (NGSP). The inter-assay coefficient of variations for HbA1c were 0.75% both at HbA1c value of 6.0% and 9.48%. eGFR was assessed by Modification of Diet in Renal Disease (MDRD) Study equation using Cr standardized to reference methods [21]. We selected the highest HbA1c, FPG, eGFR and LDL of each quarter for his/her representative HbA1c, FPG, eGFR, LDL, and then averaged the annual value of that specific year. Mean of the annual mean during the whole study period was computed thereafter. Every year’s lowest albumin and Hb were collected for the calculation of total mean albumin and Hb to prevent overestimation of these values after replacement. Mean eGFRs estimated were further classified into meaneGFR ≥60 (Stages 1–2 CKD) vs. <60 ml/min/m^2^ (stages 3–5 CKD) [22]. We defined anemia according to the definition of World Health Organization [23]. Men and women patients with mean Hb <13 and <12 g/dL, respectively, were regarded as anemia group. Hypoglycemia was identified in patients with fasting or postprandial plasma or POC glucose values <70 mg/dL [1] on any occasion during the study period.

Hyperglycemia was evaluated by mean of the annual average value of HbA1c from the index date to the last year of follow-up. These mean HbA1c were categorized into <6.0, 6.0–6.9, 7.0–7.9, 8.0–8.9, 9.0–9.9, ≥10.0%. We linked this electronic database to Taiwan’s National Death Registry, which contains information on age, sex, dates, and causes of death, for the deceased with the unique PIN. Owing to mandatory registration of all deaths in Taiwan, and all death certificates must be completed by physicians, and the mortality registry is generally accurate and complete [24,25].

The study endpoints were all-causes and cardiovascular mortality. Cardiovascular mortality was identified if the patient’s cause of death was coded with (Rheumatic heart disease [ICD-9-CM: 390–392, 393–398; ICD-10-CM: I01-I02.0, I05-I09], Ischemic heart disease [ICD-9-CM: 410–414; ICD-10-CM: I20-25], Other forms of heart disease [ICD-9-CM: 420–429; ICD-10-CM: I30-I52] or Cerebrovascular disease [ICD-9-CM: 430–438; ICD-10-CM: I60-I69).

### Statistical analysis

All-cause and cardiovascular mortality rates were calculated with person-years as the denominator under the Poisson assumption. We assessed the independent association of each level of mean HbA1c with the relative hazards of all-cause or cardiovascular mortality by conducting Cox proportional hazard regression model. We evaluated the mortality rates and relative hazards of each level of HbA1c in relation to all-cause or cardiovascular mortality according to the status of anemia, and further divided anemia status in to different mean eGFR (Stages 1–2 CKD vs. Stages 3–5 CKD). Patients with mean HbA1c 6.0–6.9% during the follow-up period was the reference group in each stratification. We used the HbA1c of 6.0–6.9% as the reference because HbA1c goal for adults is recommended to be <7.0% according to the current American Diabetes Association’s guideline [1].

The adjustment for potential confounders were made step wise. First, regression models were adjusted for type of diabetes, age, and sex (Model 1); second, in addition to the variables adjusted in Model 1, prescribed antidiabetic, antihypertensive and antilipid medications were further adjusted (Model 2). We additionally added selected comorbidities and diabetic complications together with laboratory results such as mean FPG, eGFR, LDL, albumin and Hb levels to the regression models (Model 3). All statistical analyses were performed with SAS (version 9.4; SAS Institute, Cary, NC). A p-value < 0.05 was considered statistically significant.

## Results

### Baseline patients’ characteristics

Table 1 describes the baseline characteristics of the study subjects according to different mean HbA1c. More patients with type 1 diabetes had mean HbA1c ≥8.0% while most of the patients with type 2 diabetic achieved mean HbA1c <8.0%. Patients aged <50 years kept their mean HbA1c within 7.0–8.9% while those aged >50 years had their mean HbA1c controlled around 6.0–7.9%. There was no sex difference between various mean HbA1c.

**Table 1:** Characteristics of the study subjects according to different mean HbA1c.

Variables*^,^^†^		Mean HbA1c (%)					
	<6.0	6.0–6.9	7.0–7.9	8.0–8.9	9.0–9.9	≥10	*P*
Total	1,618	12,389	15,172	8,843	4,431	3,612	
**General Characteristics**							
Type of diabetes							
Type 1 diabetes	0.37%	0.31%	0.57%	1.15%	1.99%	3.96%	
Type 2 diabetes	99.63%	99.69%	99.43%	98.85%	98.01%	96.04%	<0.0001^‡^
Mean age (±SD)	63.32±13.71	61.29±12.38	58.95±12.59	56.95±13.22	55.09±13.87	52.10±15.18	<0.0001^§^
<50 years	16.27%	17.29%	22.63%	29.87%	34.91%	43.25%	
50–69 years	49.38%	57.82%	57.20%	52.63%	49.73%	44.66%	
>69 years	34.34%	24.89%	20.16%	17.49%	15.36%	12.09%	<0.0001^‡^
Sex							
Women	44.99%	46.84%	47.34%	46.11%	45.25%	44.60%	
Men	55.01%	53.16%	52.66%	53.89%	54.75%	55.40%	0.0110^‡^
**Comorbidities and Complications**							
Coronary artery disease	32.63%	34.38%	31.75%	31.44%	30.08%	23.09%	<0.0001^‡^
Heart failure	13.54%	12.11%	11.86%	13.33%	12.93%	11.32%	0.0019^‡^
Hypertensive disease	75.46%	76.10%	75.38%	73.55%	72.49%	61.63%	<0.0001^‡^
Cerebrovascular Disease	15.51%	16.25%	15.61%	15.09%	13.86%	11.54%	<0.0001^‡^
Peripheral artery disease	4.51%	4.01%	4.90%	5.77%	5.39%	4.87%	<0.0001^‡^
Diabetic nephropathy	27.01%	24.13%	33.20%	41.33%	43.31%	38.68%	<0.0001^‡^
Diabetic retinopathy	20.46%	28.07%	37.45%	41.66%	43.83%	37.13%	<0.0001^‡^
Diabetic neuropathy	17.43%	14.94%	19.46%	23.86%	26.00%	27.57%	<0.0001^‡^
Hypoglycemia	14.22%	12.23%	20.95%	26.09%	24.37%	16.75%	<0.0001^‡^
**Laboratory results**							
**Mean**							
HbA1c (%) (±SD)	5.66±0.33	6.59±0.27	7.46±0.28	8.44±0.29	9.43±0.28	11.13±1.12	<0.0001^§^
FPG (mg/dL) (±SD)	113.84±22.89	123.55±20.01	142.10±25.97	163.29±32.66	185.78±40.58	220.90±58.40	<0.0001^§^
LDL (mg/dL) (±SD)	99.38±26.90	102.64±22.94	103.83±23.12	106.48±25.37	111.04±28.38	117.95±33.92	<0.0001^§^
eGFR (ml/min/m^2^) (±SD)	52.92±14.02	55.70±10.42	55.26±10.73	54.13±11.54	54.17±11.16	55.51±9.41	<0.0001^§^
Stages 1–2 CKD	54.27%	62.73%	60.02%	55.98%	55.91%	63.37%	
Stages 3–5 CKD	45.73%	37.27%	39.98%	44.02%	44.09%	36.63%	<0.0001^‡^
Hb (g/dL) (±SD)	12.28±2.28	13.03±2.08	12.93±2.12	12.77±2.17	12.72±2.20	12.82±2.22	<0.0001^§^
No Anemia	51.13%	66.32%	63.48%	58.58%	56.85%	57.25%	
Anemia	48.87%	33.68%	36.52%	41.42%	43.15%	42.75%	<0.0001^‡^
Albumin (g/dL) (±SD)	3.75±0.70	4.01±0.64	3.97±0.64	3.85±0.66	3.74±0.67	3.55±0.70	<0.0001^‡^
≥3.5	65.57%	80.73%	79.15%	73.34%	68.98%	56.01%	
<3.5	34.43%	19.27%	20.85%	26.66%	31.02%	43.99%	<0.0001^‡^
**Outcome**							
All-cause Mortality	41.90%	23.15%	23.41%	28.68%	31.26%	36.52%	<0.0001^‡^
Cardiovascular Mortality	7.42%	4.37%	4.78%	5.30%	6.05%	6.84%	<0.0001^‡^
Mean follow-up period (year) (±SD)	5.39±3.93	6.01±4.11	7.19±4.56	7.39±4.77	7.07±4.81	6.38±4.55	<0.0001^§^

* Data are % or mean (±SD).

^†^ eGFR: Estimated glomerular filtration rate; FPG: Fasting plasma glucose; Hb: Hemoglobin; HbA1c: Glycated hemoglobin; LDL: Low density lipoprotein; SD: Standard deviation; No anemia: Hb ≥ 13 in men and ≥12 g/dL in women; Anemia: Hb <13 in men and <12 g/dL in women.

^‡^ Based on Chi-square Method.

^§^ Based on ANOVA Method.

Generally, the prescription of oral antidiabetic agents except meglitinide and metformin became significantly higher if mean HbA1c reached ≥7.0%. Physicians’ order of insulin injection became greater if the patients’ mean HbA1c came to ≥8.0% (S1 Table). There was no considerable difference of antihypertensive medications usage or the prevalence of cardiovascular diseases among different mean HbA1c. Interestingly, hypoglycemia was more frequent in those with mean HbA1c 7.0–9.9% (20.95–26.09%) rather than in those with mean HbA1c <7.0% (12.23–14.22%).

Patients with more elevated mean HbA1c had higher mean FPG and LDL, but those with mean HbA1c <6.0% had lowest mean eGFR and Hb. The prevalence of Stages 3–5 CKD and anemia were highest in those with mean HbA1c <6.0% (45.73% and 48.87, respectively).

During the mean follow-up around 7 years, the highest percentage of all-cause and cardiovascular mortality were encountered in those with mean HbA1c <6.0% (41.90% and 7.42%, respectively) while those with mean HbA1c 6.0–7.9% had the lowest proportion of all-cause mortality (~23% and ~4%, respectively).

### Anemia- and CKD staging-specific all-cause and cardiovascular mortality rates by different mean HbA1c levels

In non-anemic patients with stages 1–2 CKD had lowest rates of all-cause and cardiovascular mortality by different HbA1c levels. The least all-cause and cardiovascular mortality rate in this group was found in those with mean HbA1c 7.0–7.9% (10.61/1,000 person-years (PY) vs. 2.44/1,000 PY, respectively), and the highest all-cause and cardiovascular mortality rates were found in mean HbA1c ≥10.0%.

If their CKD stages developed into 3–5 in non-anemic patients, all-cause mortality turned out to be more elevated. The lowest all-cause and cardiovascular mortality rates were observed in patients with mean HbA1c 7.0–7.9% while the highest rates were detected in those with mean HbA1c <6.0% or ≥10.0%.

In patients with either stages 1–2 or stages 3–5 CKD, if they were comorbid with anemia, all-cause and cardiovascular mortality rates were discovered to be excessively high. Irrespective of CKD stages, the highest all-cause mortality rates were consistently noted in anemic patients with mean HbA1c 6.0% (S3 Table), but for cardiovascular mortality, the highest mortality rates were observed in mean HbA1c <6.0 and ≥10.0%, respectively, in stages 1–2 or stages 3–5 CKD (S4 Table).

### Anemia- and CKD staging-specific relative hazards of all-cause and cardiovascular mortality by different mean HbA1c levels

Because of a significant interaction of mean HbA1c with both mean eGFR and mean Hb (P 0.007 and <0.0001, respectively), we performed a stratified analysis to estimate the CKD staging- and anemia-specific hazard ratios (HRs) of all-cause or cardiovascular mortality for various mean HbA1c levels.

**Fig 1:**
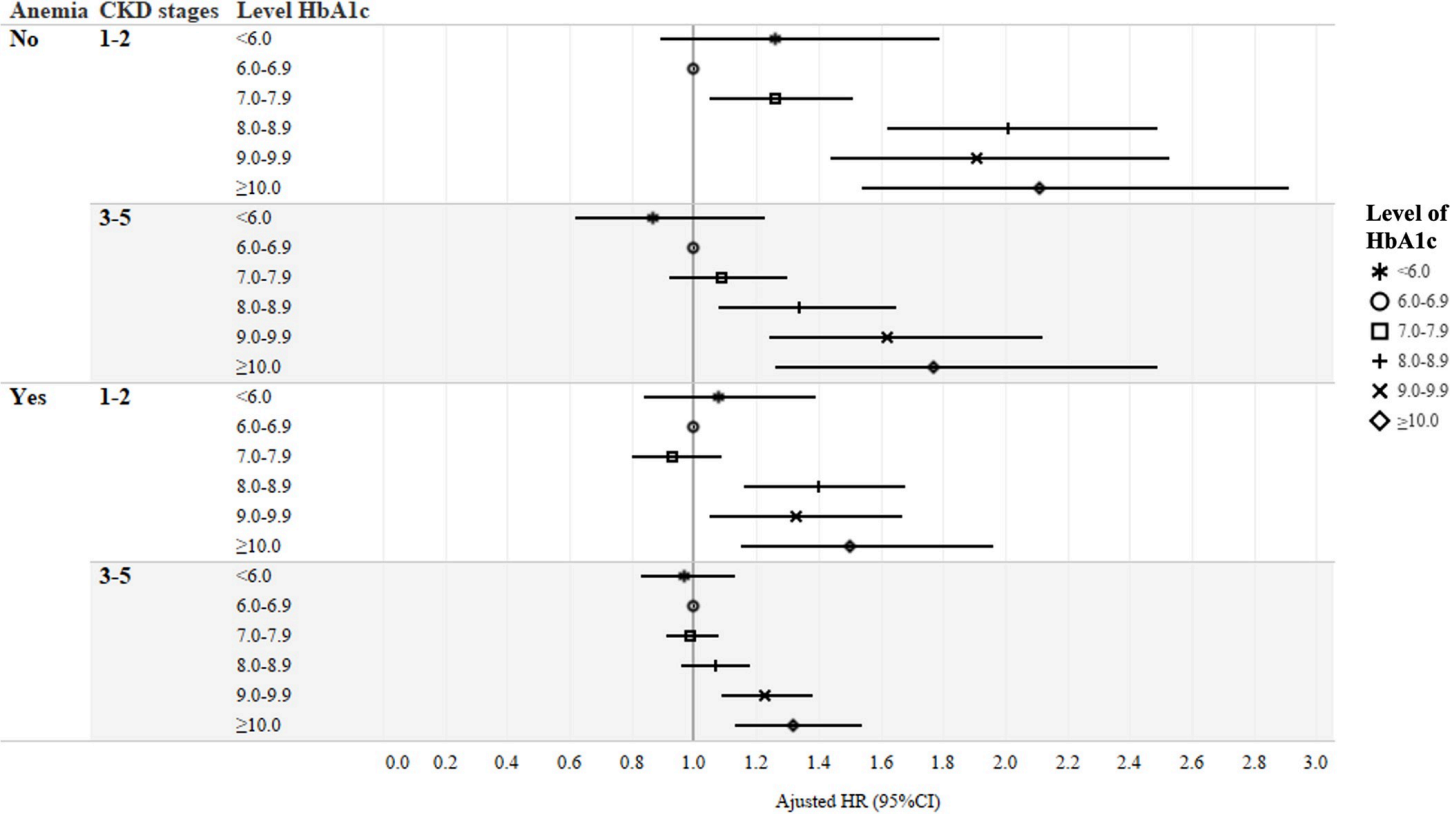
Relative hazards of all-cause mortality by different mean HbA1c levels with stratification by anemia and chronic kidney disease staging status.

In stages 1–2 CKD non-anemic patients, the lowest HR of all-cause mortality was observed in those with mean HbA1c 7.0–7.9% (HR: 0.91; 95% Confidence Interval [CI] 0.79–1.05), and higher HRs were found in those with mean HbA1c <6.0 and ≥8.0% in model 1. More elevated HRs of all-cause mortality were observed in those with higher mean HbA1c ≥7.0% after adjustment of medications in model 2. Simultaneous adjustment of comorbidities and laboratory results attenuated the HRs in model 3, and the significantly elevated mortality remained in those with mean HbA1c ≥7.0. Excessive HRs observed in those with mean HbA1c <6.0% in model 1 became inconsequential in model 2 (HR: 1.07; 95% CI 0.79–1.44) and in model 3 (HR: 1.26; 95% CI 0.89–1.79) (Fig 1 and S3 Table).

If the non-anemic patients’ CKD staging progressed into 3–5, the lowest HR was consistently observed in those with mean HbA1c 7.0–7.9% (HR: 0.83; 95% CI 0.72–0.96) while higher HRs were noted in those with mean HbA1c <6.0% and ≥9.0% in model 1. The importance of all-cause mortality in those with mean HbA1c <6.0% and 7.0–7.9% became insignificant in model 2 and 3, but those with mean HbA1c ≥8.0% showed persistently increased risk of all-cause mortality.

Similarly, if patients with stages 1–2 and 3–5 CKD became coexisted with anemia, the extra risk observed in those with mean HbA1c <6.0% in model 1 became inconsequential in model 2 and 3. Higher risks of all-cause mortality were persistently noted in those patients with mean HbA1c ≥8.0 in stages 1–2 CKD, and mean HbA1c ≥ 9.0% in stages 3–5 CKD.

**Fig 2:**
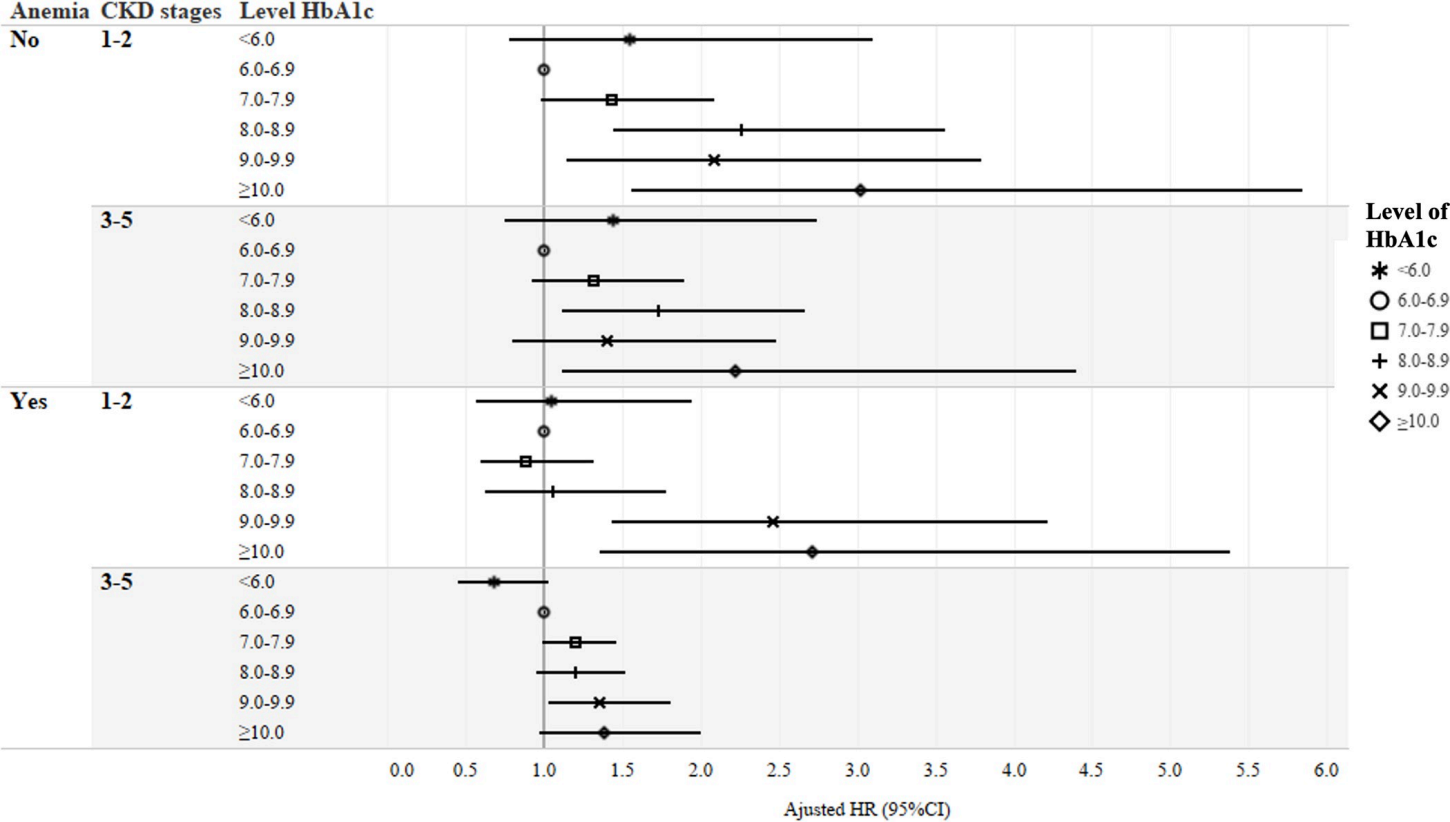
Relative hazards of cardiovascular mortality by different mean HbA1c levels with stratification by anemia and chronic kidney disease staging status.

Low HbA1c <6.0% was not related to increased risk of cardiovascular mortality in either anemic status or CKD staging in all 3 models. Persistent elevated risks of cardiovascular mortality were noted in non-anemic patients of mean HbA1c ≥8.0% in stages 1–2 CKD, mean HbA1c ≥8.0–8.9% and ≥10.0% in stages 3–5 CKD. If there were coexisting anemia, higher risks of cardiovascular mortality were noted in those with mean HbA1c ≥9.0% or ≥9.0–9.9% in stages 1–2 and 3–5 CKD, respectively (Fig 2 and S4 Table).

## Discussion

In our study, excessive risks of all-cause mortality discovered in those with mean HbA1c <6.0% became insignificant after adjustment for medications, co-morbidities and laboratory results. Low mean HbA1c (<6.0%) was not associated with increased cardiovascular mortality in either anemic status or CKD staging. Overall all-cause and cardiovascular mortality of the diabetes subjects from our tertiary medical center were 41.5 and 7.9/1,000 PY which were much higher than 6 and 0.9/1,000 PY of general population of Taiwan [26].

In the UKPDS study, the lowest mortality rate was seen in those with mean HbA1c <6.0%, while the highest one was observed in those with mean HbA1c ≥10.0% [3]. In our study, however, the highest overall all-cause mortality rate was seen in those with mean HbA1c <6.0% in anemic group. Higher prevalence of anemia and stages 3–5 CKD in those with mean HbA1c <6.0% compared to all other mean HbA1c in our study might have increased the risk of all-cause mortality [27–29]. The details of the prevalence of anemia and CKD were unavailable in the UKPDS study [3]. In the Israel study, in which the prevalence of CKD was also more prevalent in those with average HbA1c <6.5% [30], higher all-cause mortality rate was noticed in that HbA1c <6.5% group comparable to the results from our study. In the observation study of 587 UK primary care practices [7], all-cause mortality rates were high if patients’ mean HbA1c <6.0% or ≥8.0% like the results from our study. Because they recruited only patients with diabetes aged ≥70 years, their mortality rates were much higher than those of non-anemic patients of our study, but comparable to those with anemic patients of ours. In the NHANES III analysis [31], cardiovascular mortality rates were the highest among patients of diabetes if their baseline HbA1c were 6.0–6.9% (32.6/ 1,000 PY). On the contrary, cardiovascular mortality were highest in those with mean HbA1c <6.0% or ≥10.0% in our study, but mortality rates were much lower than those of NHANES III irrespective of anemia status or CKD staging. We summarized the rates and relative hazards of all-cause and cardiovascular mortality of previous studies in Table 2.

**Table 2:** Literature association between HbA1c and all-cause and cardiovascular mortality.

Study	No. of subjects	DM*/ DKD* status	Age (years)	Follow- up (years)	HbA1c (%) categories	All-cause mortality				Cardiovascular mortality	
						Rates (/1000 PY*)		Hazard Ratio (95%CI*)		Rates (/1000 PY*)	Hazard Ratio (95%CI*)
Stratton et al. 2000 [3]	3,642	T2DM	53±8 (Mean)	7.5–12.5	<6	17.00 (13.10–22.00)					
					6-<7	23.30 (18.50–29.20)					
					7-<8	30.00 (23.80–37.70)					
					8-<9	31.80 (24.70–40.80)					
					9-<10	37.00 (27.30–50.20)					
					≥10	40.70 (26.50–64.50)					
Saydah et al. 2009 [31]	1,455	DM	≥20	6–12	<6.0	35.10 (19.30–72.90)		1.00		11.00 (5.10–21.00)	1.00
					6.0-<7	58.60 (40.20–137.40)		1.00 (0.60–1.80)		32.60 (18.90–69.60)	1.90 (1.00–3.70)
					7.0-<8	58.80 (37.00–131.40)		1.10 (0.60–2.10)		15.90 (5.40–26.60)	1.10 (0.40–2.70)
					≥8	55.74 (35.61–125.54)		1.70 (1.00–2.70)		23.00 (13.30–125.50)	2.50 (1.10–5.60)
Currie et al. 2010 [4]	27,965	T2DM	≥50	4.5±2.7 (Mean)	*D1(6.4)			1.79 (1.45–2.22)			
					D2(6.9)			1.45 (1.17–1.80)			
					D3(7.3)			1.35 (1.09–1.67)			
					D4(7.5)			1.00			
					D5(7.8)			0.98 (0.79–1.21)			
					D6(8.1)			1.15 (0.95–1.41)			
					D7(8.4)			1.21 (1.00–1.48)			
					D8(8.9)			1.21 (0.99–1.47)			
					D9(9.4)			1.46 (1.21–1.77)			
					D10(10.6)			1.80 (1.49–2.17)			
Shurraw et al. 2011 [33]	23,296	DKD Stage 3	65.9–73.0 ±10.6–13.3	4	<7			1.0			
				(Median)	7–9			1.04 (0.96–1.13)			
					>9			1.35 (1.20–1.53)			
		DKD Stage 4			<7			1.0			
					7–9			1.03 (0.87–1.21)			
					>9			1.39 (1.10–1.76)			
Skriver et al. 2012 [6]	17,760	T2DM	65–67 (Mean)	2 (Median)	<5			1.75 (1.14–2.69)			
					5.0–5.9			1.12 (0.97–1.29)			
					6.0–6.9			1.00			
					7.0–7.9			1.22 (1.08–1.38)			
					8.0–8.9			1.37 (1.18–1.60)			
					≥9			1.44 (1.24–1.66)			
Ramirez et al. 2012 [34]	9,201	DKD (Hemo-dialysis)	59–66.6	1.4	<5			1.35 (1.09–1.67)			
			(Mean)		5–5.9			1.18 (1.01–1.37)			
					6–6.9			1.21 (1.05–1.41)			
					7–7.9			1.0			
					8–8.9			1.16 (0.94–1.43)			
					≥9			1.38 (1.11–1.71)			
Twito et al. 2013 [30]	2,994	DM (New onset)	≥65	5.3–5.8 (Mean)	<6.5	74.30		1.40 (1.20–1.60)			
					6.5–6.99	54.70		1.00			
					7–7.49	65.20		1.20 (0.96–1.50)			
					≥7.5	86.10		1.60 (1.30–1.90)			
Schöttker et al. 2016 [17]											
	5,778	No DM	67.9±11.0	11.4±4.9	<5.0			1.08 (0.95–1.23)			1.11 (0.91–1.35)
			(Mean)	(Mean)							
Li W et al. 2016 [8]	35,261	T2DM	30–94	8.7 (Mean)	<6.0	61.67		1.27 (1.14–1.42)			
					6.0–6.9	81.94		1.00			
					7.0–7.9	59.96		0.96 (0.86–1.08)			
					8.0–8.9	39.75		1.03 (0.89–1.18)			
					9.0–9.9	27.06		1.19 (1.01–1.40)			
					10.10.9	17.48		1.52 (1.26–1.83)			
					≥11%	18.02		1.64 (1.35–1.99)			
Forbes et al. 2018 [7]	54,803	DM	≥70	5	3.0-<6.0	96.00^†^	95.00^‡^	1.22 (1.11–1.34)^†^	1.15 (1.04–1.27)^‡^		
					6.0–6.5	80.00^†^	72.00^‡^	1.08 (1.00–1.17)^†^	0.98 (0.90–1.06)^‡^		
					6.5-<7.0	74.00^†^	64.00^‡^	1.06 (0.99–1.14)^†^	0.97(0.90–1.04)^‡^		
					7.0–7.5	69.00^†^	62.00^‡^	1.00	1.00		
					7.5-<8.0	76.00^†^	68.00^‡^	1.03 (0.95–1.11) ^†^	1.03 (0.94–1.12)^‡^		
					8.0-<8.5	89.00^†^	83.00^‡^	1.19 (1.08–1.30) ^†^	1.17 (1.07–1.29)^‡^		
					8.5-<9.0	94.00^†^	82.00^‡^	1.21 (1.08–1.35) ^†^	1.19 (1.06–1.33)^‡^		
					9.0-<9.5	107.00^†^	104.00^‡^	1.37 (1.20–1.56) ^†^	1.44 (1.27–1.63)^‡^		
					9.5-<10	126.00^†^	101.00^‡^	1.47 (1.23–1.75) ^†^	1.33 (1.11–1.59) ^†^		
					≥10	144.00^†^	141.00^‡^	1.82 (1.57–2.10) ^†^	1.98 (1.71–2.29)^‡^		
Li FR et al. 2019 [5]	3,824	DM	≥50	5.8 (Median)	<5.40			1.66 (1.19–2.33)			
					5.40–5.60			1.07 (0.74–1.55)			
					>5.60-<7.37			1.00			
					7.37–8.43			1.25 (0.99–1.58)			
					>8.43			1.40 (1.09–1.80)			
Raghavan et al. 2019 [9]	329,624	DM	65.6±10.7 (Mean)	2–5	<6.0	50.80 (50.00–51.60)		1.14 (1.11–1.18)		16.60 (16.20–17.10)	1.08 (1.02–1.13)
					6.0–6.9	47.10 (46.60–47.50)		1.00		15.90 (15.70–16.20)	1.00
					7.0–7.9	48.50 (48.10–49.00)		1.06 (1.03–1.08)		17.20 (16.90–17.50)	1.11 (1.08–1.14)
					8.0–8.9	52.60 (51.90–53.30)		1.18 (1.14–1.21)		19.10 (18.60–19.50)	1.25 (1.22–1.29)
					≥9	60.00 (59.20–60.90)		1.45 (1.41–1.49)		22.40 (21.90–23.00)	1.52 (1.48–1.56)
Limkunakul et al. 2019 [32]	618	DKD	60	4.2	<7	65±8.7		1.0			
		Stage 3	(Mean)	(Median)	7–8	38±8.6		1.40 (0.88–2.20)			
					≥8	45±9.0		1.15 (0.73–1.80)			
Inoue et al. 2021 [18]	39,453	No DM	≥20	5	4-<5			1.30 (1.16–1.48)			1.17 (0.80–1.59)
				10	4-<5			1.12 (1.03–1.22)			1.21 (0.92–1.54)

* CI: Confidence interval; D: Decile; DKD: Diabetic Kidney Disease; DM: Diabetes Mellitus; PY: Person-years; T2DM: Type 2 Diabetes Mellitus

^†^ Male

^‡^ Female.

Information about mortality rates in patients with CKD was scarce in the literature. In a nephrology clinic-based study, University of Washington, Seattle, USA [32], of the 618 patients with eGFR <60 ml/ min/m^2^, 232 patients died in a median follow-up of 5 years with mortality rate of 64.8/ 1,000 PY, higher than those of non-anemic stage 1–5 CKD, comparable with those of anemic stage 1–2 CKD, but lower than those of anemic stages 3–5 CKD.

Previous studies showed a U-shape [4–6] or J-shape [7–9] relationship between HbA1c and the risks of all-cause mortality, and lower A1c was associated with reduced survival even after adjustment of confounders including creatinine function [4], CKD staging [7,8] or admission for nephropathy [6]. To avoid the possibility of underestimation of low HbA1c level in the status of advanced CKD and anemia, we evaluated the risks of all-cause mortality in different anemia status and CKD staging. After such stratification, we found out that increased mortality observed in low mean HbA1c <6.0% became insignificant after adjustment of medications, comorbidities, and laboratory statuses in either CKD staging or anemia status in contrast to previous studies with no CKD staging [4–9], patients with diabetes in stages 3–4 CKD [33] or those undergoing hemodialysis [34].

In the study of US Veterans Affairs Healthcare system [9], a J-shaped association between baseline HbA1c and short-term cardiovascular mortality. In the Health and Retirement Study [5], there was a trend toward U-shaped association with baseline HbA1c and cardiovascular mortality, but with little statistical significance. With stratification of CKD staging and anemia status, our study did not reveal higher risks of cardiovascular mortality in lower mean HbA1c <6.0%.

Kalantar-Zadeh et al., analyzed 23,618 patients with diabetes undergoing maintenance hemodialysis in DaVita outpatient clinics between 2001 and 2004 [35] with subgroup analysis in anemia status. Although lower HbA1c values appeared to be associated with higher mortality rates after adjusting potential confounders, higher HbA1c values were incrementally associated with increased death risks in non-anemic patients whereas in anemic patients the association did not show the same pattern. Kuo et al., also studied the effect of Hb on the predictive value of HbA1c in 1,558 diabetic patients with stages 3–4 CKD in Kaohsiung Medical University in Southern Taiwan [36]. In their analysis, higher quartiles of HbA1c was associated with a higher risk of all-cause mortality in patients with Hb ≥10 g/ dL, but not in those with Hb <10 g/dL. In our study, however, higher risks observed in those with HbA1c <6.0% became insignificant after adjustment of medications, comorbidities, and laboratory results despite the status of anemia and CKD staging. Although above two studies adjusted surrogates of nutritional status and inflammation [35,36], they did not adjusted medications use and their follow-up periods were relatively not long enough compared to that of ours.

In a meta-analysis of six population-based cohort studies from Europe and the USA [17], Schöttker et al., reported that in non-diabetic older adults aged ≥50 years, the observed effect estimates for HbA1c 6.0–6.5% were strongly attenuated by adjustment for smoking, C-reactive protein, and renal function. In NHANES, the only study that examined iron deficiency status in that meta-analysis, a pronounced J-shaped association of very low HbA1c levels with all-cause mortality lost its statistical significance with adjustment of race/ ethnicity, alcohol consumption, BMI as well as biomarkers of iron deficiency anemia and liver function [17]. The authors concluded that the strongest confounder was anemia because there is a close relationship between Hb and HbA1c levels [37].

Anemia of CKD is a multifactorial process due to relative EPO deficiency, uremic-induced inhibitors of erythropoiesis, shortened erythrocyte survival, and disordered iron homeostasis [38]. A shortened RBC life span was evident even in early-stage CKD patients. RBC lifespan durations in CKD stages 1–5 were 122±50, 112±26, 90±32, 88±28, and 60±24 days, respectively [39]. Although patients with autosomal dominant polycystic kidney disease could maintain their Hb levels up to CKD stages 3–4 [40], in patients with chronic interstitial nephritis [41,42] or diabetic nephropathy [43], anemia tends to occur at an earlier stage. Treatment of anemia in patients with CKD using iron replacement therapy resulting in lowering of the A1c values secondary to the formation of new erythrocytes in the bloodstream, causing a change of proportion of young to old cells, and also from an alteration in the RBC glycation rates [44,45]. Sato et al., analyzed 62,931 Japanese people dividing into six groups in accordance with their eGFR and Hb levels. They reported that independent of eGFR levels, anemic people had significantly higher mortality with odds ratio of 2.25 (95% CI 1.89–2.67) after using propensity score matching [46]. In a subset analysis of the Atherosclerosis Risk in Communities study of 941 diabetic people with varying degrees of CKD, the authors found out that the correlation between HbA1c and fasting glucose weakens as renal function worsens. Among those diabetic people with neither anemia nor CKD, the correlation coefficient between HbA1c and fasting glucose was r = 0.70, compared with r = 0.35 among those with both anemia and severe CKD [47]. Consequently, interpretation of low HbA1c associated high mortality in patients with diabetes should be cautious as low HbA1c in these patients does not necessarily equal to strict diabetic control. Fructosamine, a measure of glucose binding to total serum proteins [48], is thought to negate the effect of reduced red blood cell survival times, but Jung et al. reported that the strength of association between fructosamine with fasting glucose (r = 0.48) was not stronger than the HbA1c and fasting glucose relationship (r = 0.70); and the weaker association between fructosamine and fasting glucose was more pronounced in people with anemia than in people without anemia regardless of CKD category [47].

Our study has several methodological strengths. First, because patients with diabetes were retrieved from the interlinkage with the FEMH hospital electronic medical records, the possibility for selection bias arising from non-response or loss of the follow-up was considered small. In addition, the attainment of disease information from the hospital database rather than self-reports might largely reduce the possibility of information bias. Second, one of the potential advantages of using hospital datasets in clinical research is the easy access to the longitudinal records for a relatively large sample of patients [49]. Third, we only recruited patients with the usage of oral or parenteral antidiabetic agents which might have reduced the disease misclassification bias in our study. Fourth, we could identify several cardiovascular risk factors, comorbidities, complications, medications and laboratory results which might have affected the survival of our patients. Most importantly, we could classify anemia and CKD staging in our analysis which might have diminished the effect of low HbA1c contributed by anemia or CKD.

In spite of the above strengths, our study has several limitations. A number of factors not detected in our study including BMI, smoking, and blood pressure which might have provided residual confounding in our study. However, we have already adjusted several cardiovascular risk factors, comorbidities, medications and laboratory results in the models. We were also limited by not having any specific data for important indicators such as frailty, cognitive function and functional status which were crucial in the analysis of mortality. In addition, this study was based on a single tertiary medical center, and the baseline characteristics of our study participants might have been different from general diabetic population.

## Conclusion

In conclusions, with stratification of anemia and CKD staging, there was insignificant association between lower mean HbA1c and all-cause or cardiovascular mortality observed in our study. Interpretation of low HbA1c related increased mortality should be cautious in patients with diabetes with careful consideration of underlying comorbidities.

## Supporting information

S1 Table Medications use in the study subjects according to different mean HbA1c. * Data are % or mean (±SD). ^†^ eGFR: Estimated glomerular filtration rate; FPG: Fasting plasma glucose; Hb: Hemoglobin; HbA1c: Glycated hemoglobin; LDL: Low density lipoprotein; SD: Standard deviation; No anemia: Hb ≥ 13 in men and ≥12 g/dL in women; Anemia: Hb <13 in men and <12 g/dL in women. ^‡^ Based on Chi-square Method. ^§^ Based on ANOVA Method.
(DOCX)

S2 Table Type of diabetes and its comorbidities and complications collected in this study. (DOCX)

S3 Table Overall rates and relative hazards of all-cause mortality by different mean HbA1c levels with stratification by anemia and chronic kidney disease staging status. * Inconsistency between total population and population summed for individual variable was due to missing information; HbA1c = glycated hemoglobin; CKD = chronic kidney disease; No anemia: Hb ≥ 13 in men and ≥12 g/dL in women; Anemia: Hb <13 in men and <12 g/dL in women. ^†^ Based on Poisson assumption, CI = confidence interval. ^‡^ HR = hazard ratio; CI = confidence interval. ^§^ p values for the interaction of mean HbA1c with mean hemoglobin and mean estimated glomerular filtration rate were < 0.0001 and 0.0007, respectively. ^||^ Based on Cox proportional hazard regression with adjustment for general characteristics (i.e., type of diabetes, age and sex). ^¶^ Based on Cox proportional hazard regression with adjustment for the general characteristics adjusted in Model 1 plus antidiabetic, antihypertensive, and antilipid medications. ^#^ Based on Cox proportional hazard regression with all covariates included in Model 2 plus comorbidities, complications, and laboratory results.
(DOCX)

S4 Table Overall rates and relative hazards of all-cause mortality by different mean HbA1c levels with stratification by anemia and chronic kidney disease staging status. * Inconsistency between total population and population summed for individual variable was due to missing information; HbA1c = glycated hemoglobin; CKD = chronic kidney disease; No anemia: Hb ≥ 13 in men and ≥12 g/dL in women; Anemia: Hb <13 in men and <12 g/dL in women. ^†^ Based on Poisson assumption, CI = confidence interval. ^‡^ HR = hazard ratio; CI = confidence interval. ^§^ p values for the interaction of mean HbA1c with mean hemoglobin and mean estimated glomerular filtration rate were 0.2860 and 0.0022, respectively. ^||^ Based on Cox proportional hazard regression with adjustment for general characteristics (i.e., type of diabetes, age and sex). ^¶^ Based on Cox proportional hazard regression with adjustment for the general characteristics adjusted in Model 1 plus antidiabetic, antihypertensive, and antilipid medications. ^#^ Based on Cox proportional hazard regression with all covariates included in Model 2 plus comorbidities, complications, and laboratory result.
(DOCX)
