# Supplementary material for: Low mean HbA1c does not increase all-cause and cardiovascular mortality in patients with diabetes: Effect-modifications by anemia and chronic kidney disease stages
Source: PLoS One. 2022 Aug 11;17(8):e0272137. doi: 10.1371/journal.pone.0272137 (PMC9371313; doi:10.1371/journal.pone.0272137)
Supplement: S1 Table — * Data are % or mean (±SD). † eGFR: Estimated glomerular filtration rate; FPG: Fasting plasma glucose; Hb: Hemoglobin; HbA1c: Glycated hemoglobin; LDL: Low density lipoprotein; SD: Standard deviation; No anemia: Hb ≥ 13 in men and ≥12 g/dL in women; Anemia: Hb <13 in men and <12 g/dL in women. ‡ Based on Chi-square Method. § Based on ANOVA Method. (DOCX) [file pone.0272137.s001.docx]

S1 Table. Medications use in the study subjects according to different mean HbA1c

| Variables^*,†^ |  |  | | Mean HbA1c (%) | | | | | |
| --- | --- | --- | --- | --- | --- | --- | --- | --- | --- |
|  |  | <6.0 | 6.0-6.9 | | 7.0-7.9 | 8.0-8.9 | 9.0-9.9 | ≥10 | *P* |
| Total |  | 1,618 | 12,389 | | 15,172 | 8,843 | 4,431 | 3,612 |  |
| **Antidiabetic Medications** |  |  |  | |  |  |  |  |  |
| Sulphonylurea |  | 43.70% | 47.22% | | 76.22% | 84.60% | 82.92% | 79.40% | <0.0001^‡^ |
| Meglitinide |  | 21.69% | 16.00% | | 20.57% | 22.67% | 22.03% | 18.52% | <0.0001^‡^ |
| Metformin |  | 70.02% | 84.21% | | 88.19% | 88.15% | 86.68% | 85.24% | <0.0001^‡^ |
| Thiazolidinediones |  | 7.23% | 9.68% | | 21.19% | 27.90% | 30.15% | 24.47% | <0.0001^‡^ |
| Alpha-glucosidase inhibitors |  | 9.89% | 9.98% | | 18.00% | 24.61% | 24.33% | 20.99% | <0.0001^‡^ |
| Dipeptidyl peptidase-4 inhibitor |  | 21.51% | 36.39% | | 57.40% | 56.73% | 48.97% | 36.30% | <0.0001^‡^ |
| Sodium-glucose cotransporter-2 inhibitors |  | 2.04% | 5.79% | | 12.04% | 11.96% | 9.98% | 7.20% | <0.0001^‡^ |
| Insulin |  | 13.23% | 10.81% | | 25.11% | 48.77% | 61.25% | 67.88% | <0.0001^‡^ |
| Glucagon-like peptide-1 receptor agonist |  | 0.56% | 0.41% | | 1.29% | 3.10% | 3.09% | 2.93% | <0.0001^‡^ |
| **Antihypertensives** |  |  |  | |  |  |  |  |  |
| Angiotensin Converting Enzyme Inhibitors |  | 18.60% | 16.20% | | 20.82% | 22.64% | 23.02% | 19.32% | <0.0001^‡^ |
| Angiotensin Receptor Blockers |  | 57.73% | 63.48% | | 65.15% | 64.63% | 62.99% | 51.61% | <0.0001^‡^ |
| Beta-Blockers |  | 48.45% | 51.24% | | 48.17% | 45.66% | 45.23% | 36.21% | <0.0001^‡^ |
| Calcium-Channel Blockers |  | 58.22% | 59.40% | | 57.12% | 55.07% | 53.06% | 41.64% | <0.0001^‡^ |
| Diuretics |  | 50.31% | 46.36% | | 48.24% | 50.23% | 50.62% | 42.47% | <0.0001^‡^ |
| **Antilipids** |  |  |  | |  |  |  |  |  |
| Statin |  | 44.38% | 67.35% | | 71.66% | 71.04% | 68.97% | 60.71% | <0.0001^‡^ |
| Fibrates |  | 14.52% | 16.85% | | 22.07% | 26.07% | 26.36% | 23.70% | <0.0001^‡^ |

^*^ Data are % or mean (±SD)

^†^ eGFR: estimated glomerular filtration rate; FPG: fasting plasma glucose; Hb: hemoglobin; HbA1c: glycated hemoglobin; LDL: low density lipoprotein; SD: standard deviation; No anemia: Hb ≥ 13 in men and ≥12 g/dL in women; Anemia: Hb <13 in men and <12 g/dL in women.

^‡^ Based on Chi-square Method

^§^ Based on ANOVA Method
