# Supplementary material for: Low mean HbA1c does not increase all-cause and cardiovascular mortality in patients with diabetes: Effect-modifications by anemia and chronic kidney disease stages
Source: PLoS One. 2022 Aug 11;17(8):e0272137. doi: 10.1371/journal.pone.0272137 (PMC9371313; doi:10.1371/journal.pone.0272137)
Supplement: S2 Table — (DOCX) [file pone.0272137.s002.docx]

S2 Table: Type of diabetes and its comorbidities and complications collected in this study

| Diseases Classification |  | ICD-9 | ICD-10 |
| --- | --- | --- | --- |
| Diabetes | Type 1 diabetes  Type 2 diabetes | 250.x1, 250.x3  250.x0  250.x2 | E10  E11 |
| Coronary artery disease  Heart failure  Hypertensive disease | Acute myocardial infarction  Other acute and subacute forms of ischemic heart disease  Angina pectoris  Chronic ischemic heart disease  Heart failure  Hypertensive disease | 410  411  413  414  428  401-405 | I21, I22, I23  I24  I20  I25  I50  I10-I16 |
| Cerebrovascular Disease | Ischemic stroke | 433–436, 437.0, 437.1 | I63-I66, I67.2, I67.3, I67.6, I67.81, 167.82, 167.9 |
| Peripheral artery disease | Atherosclerosis (of arteries, arterioles, and capillaries)  Diabetes with peripheral circulatory disorders  Peripheral angiopathy in diseases classified elsewhere | 440  250.7  443.81 | I70  E11.5 |
| Chronic microvascular diabetic complications | Diabetes with renal manifestations  Chronic renal failure  Renal failure, unspecified | 250.4,  585  586 | E11.2  N18  N19 |
|  | Diabetes with ophthalmic manifestations  Diabetic retinopathy  Other background retinopathy and retinal vascular changes  Cystoid macular degeneration  (Cystoid macular edema)  Retinal hemorrhage  Retinal exudates and deposits  Retinal edema  Retinal ischemia  Retinal detachment and defects  Blindness and low vision  Vitreous hemorrhage | 250.5  362.0  362.1  362.53  362.81  362.82  362.83  362.84  361  369  379.23 | E11.3  H35.0  H35.35  H35.6  H35.89, H35.9  H35.81  H35.82  H33  H54  H43.1 |
|  | Diabetes with neurological manifestations  Polyneuropathy in diabetes  Idiopathic peripheral autonomic neuropathy  Carotid sinus syncope or syndrome  Peripheral autonomic neuropathy in disorders classified elsewhere  Mononeuritis of upper limb and mononeuritis multiplex  Mononeuritis of lower limb and unspecified site  Unspecified hereditary and idiopathic peripheral neuropathy  Myasthenic syndromes in diseases classified elsewhere  Orthostatic hypotension  Gastroparesis  Functional diarrhea  Neurogenic bladder NOS  Arthropathy associated with neurological disorders, Charcôt's joint  Injury to oculomotor nerve,  Injury to trochlear nerve  Injury to abducens nerve | 250.6  357.2  337.0  337.1  354  355  356.9  358.1  458.0  536.3  564.5  596.54  713.5  951.0  951.1  951.3 | E11.4  G90.0  G90.01  G90.8, G90.9  G99.0  G56  G57  G60.9  G73.3  I95.1  K31.84  K59.1  N31.9  E11.61  M14.6  S04.1  S04.2  S04.4 |
