# Supplementary material for: Low mean HbA1c does not increase all-cause and cardiovascular mortality in patients with diabetes: Effect-modifications by anemia and chronic kidney disease stages
Source: PLoS One. 2022 Aug 11;17(8):e0272137. doi: 10.1371/journal.pone.0272137 (PMC9371313; doi:10.1371/journal.pone.0272137)
Supplement: S3 Table — * Inconsistency between total population and population summed for individual variable was due to missing information; HbA1c = glycated hemoglobin; CKD = chronic kidney disease; No anemia: Hb ≥ 13 in men and ≥12 g/dL in women; Anemia: Hb <13 in men and <12 g/dL in women. † Based on Poisson assumption, CI = confidence interval. ‡ HR = hazard ratio; CI = confidence interval. § p values for the interaction of mean HbA1c with mean hemoglobin and mean estimated glomerular filtration rate were < 0.0001 and 0.0007, respectively. || Based on Cox proportional hazard regression with adjustment for general characteristics (i.e., type of diabetes, age and sex). ¶ Based on Cox proportional hazard regression with adjustment for the general characteristics adjusted in Model 1 plus antidiabetic, antihypertensive, and antilipid medications. # Based on Cox proportional hazard regression with all covariates included in Model 2 plus comorbidities, complications, and laboratory results. (DOCX) [file pone.0272137.s003.docx]

S3 Table: Overall rates and relative hazards of all-cause mortality by different mean HbA1c levels with stratification by anemia and chronic kidney disease staging status

| Level of mean HbA1c (%)^*^ | All-cause mortality | | |  | Model 1  Adjusted HR (95% CI)^‡^ |  | Model 2  Adjusted HR (95% CI)^‡^ |  | Model 3  Adjusted HR (95% CI)^‡,§^ |
| --- | --- | --- | --- | --- | --- | --- | --- | --- | --- |
|  | No. of patients | No. of mortality | Rates (per 1,000 patient-years)  (95% CI)^†^ |  |  |  |  |  |  |
| **No Anemia** |  |  |  |  |  |  |  |  |  |
| Stages 1-2 CKD |  |  |  |  |  |  |  |  |  |
| <6.0 | 442 | 51 | 20.55 (14.91-26.19) |  | 1.58 (1.18-2.12)^\|\|^ |  | 1.07 (0.79-1.44)^¶^ |  | 1.26 (0.89-1.79)^#^ |
| 6.0-6.9 | 5,064 | 343 | 11.59 (10.36-12.82) |  | 1.0 (Reference) |  | 1.0 (Reference) |  | 1.0 (Reference) |
| 7.0-7.9 | 5,593 | 418 | 10.61 (9.60-11.63) |  | 0.91 (0.79-1.05)^\|\|^ |  | 1.35 (1.16-1.57)^¶^ |  | 1.26 (1.05-1.51)^#^ |
| 8.0-8.9 | 2,858 | 328 | 15.87 (14.15-17.58) |  | 1.58 (1.35-1.84)^\|\|^ |  | 2.51 (2.12-2.98)^¶^ |  | 2.01 (1.62-2.49)^#^ |
| 9.0-9.9 | 1,354 | 152 | 15.79 (13.28-18.31) |  | 1.70 (1.40-2.06)^\|\|^ |  | 2.38 (1.92-2.94)^¶^ |  | 1.91 (1.44-2.53)^#^ |
| ≥10.0 | 1,099 | 185 | 24.37 (20.86-27.88) |  | 3.02 (2.51-3.63)^\|\|^ |  | 3.49 (2.83-4.29)^¶^ |  | 2.11 (1.54-2.91)^#^ |
| Stages 3-5 CKD |  |  |  |  |  |  |  |  |  |
| <6.0 | 183 | 49 | 38.68 (27.85-49.51) |  | 1.53 (1.13-2.06)^\|\|^ |  | 0.89 (0.65-1.21)^¶^ |  | 0.87 (0.62-1.23)^#^ |
| 6.0-6.9 | 1,854 | 341 | 25.72 (22.99-28.45) |  | 1.0 (Reference) |  | 1.0 (Reference) |  | 1.0 (Reference) |
| 7.0-7.9 | 2,339 | 419 | 20.37 (18.42-22.32) |  | 0.83 (0.72-0.96)^\|\|^ |  | 1.15 (0.99-1.34)^¶^ |  | 1.09 (0.92-1.30)^#^ |
| 8.0-8.9 | 1,316 | 304 | 24.98 (22.18-27.79) |  | 0.99 (0.84-1.15)^\|\|^ |  | 1.52 (1.27-1.81)^¶^ |  | 1.34 (1.08-1.65)^#^ |
| 9.0-9.9 | 619 | 161 | 27.83 (23.53-32.13) |  | 1.37 (1.13-1.66)^\|\|^ |  | 2.05 (1.66-2.54)^¶^ |  | 1.62 (1.24-2.12)^#^ |
| ≥10.0 | 359 | 123 | 38.75 (31.90-45.60) |  | 2.08 (1.69-2.57)^\|\|^ |  | 2.75 (2.18-3.46)^¶^ |  | 1.77 (1.26-2.49)^#^ |
| **Anemia** |  |  |  |  |  |  |  |  |  |
| Stages 1-2 CKD |  |  |  |  |  |  |  |  |  |
| <6.0 | 202 | 109 | 98.37 (79.90-116.84) |  | 1.37 (1.11-1.69)^\|\|^ |  | 1.26 (1.02-1.56)^¶^ |  | 1.08 (0.84-1.39)^#^ |
| 6.0-6.9 | 1,231 | 428 | 59.62 (53.97-65.27) |  | 1.0 (Reference) |  | 1.0 (Reference) |  | 1.0 (Reference) |
| 7.0-7.9 | 1,465 | 498 | 49.68 (45.32-54.05) |  | 0.82 (0.72-0.93)^\|\|^ |  | 1.05 (0.92-1.20)^¶^ |  | 0.93 (0.80-1.09)^#^ |
| 8.0-8.9 | 851 | 341 | 58.51 (52.30-64.72) |  | 1.07 (0.93-1.24)^\|\|^ |  | 1.52 (1.31-1.77)^¶^ |  | 1.40 (1.16-1.68)^#^ |
| 9.0-9.9 | 462 | 197 | 61.11 (52.58-69.64) |  | 1.20 (1.01-1.43)^\|\|^ |  | 1.52 (1.27-1.82)^¶^ |  | 1.33 (1.05-1.67)^#^ |
| ≥10.0 | 413 | 199 | 75.21 (64.76-85.65) |  | 1.81 (1.53-2.16)^\|\|^ |  | 1.96 (1.62-2.38)^¶^ |  | 1.50 (1.15-1.96)^#^ |
| Stages 3-5 CKD |  |  |  |  |  |  |  |  |  |
| <6.0 | 397 | 242 | 104.63 (91.45-117.82) |  | 1.34 (1.16-1.53)^\|\|^ |  | 1.06 (0.92-1.22)^¶^ |  | 0.97 (0.83-1.13)^#^ |
| 6.0-6.9 | 2,238 | 1,225 | 78.36 (73.97-82.75) |  | 1.0 (Reference) |  | 1.0 (Reference) |  | 1.0 (Reference) |
| 7.0-7.9 | 3,061 | 1,637 | 64.56 (61.43-67.69) |  | 0.80 (0.74-0.86)^\|\|^ |  | 1.02 (0.94-1.10)^¶^ |  | 0.99 (0.91-1.08)^#^ |
| 8.0-8.9 | 2,070 | 1,157 | 64.39 (60.68-68.10) |  | 0.89 (0.82-0.97)^\|\|^ |  | 1.15 (1.06-1.26)^¶^ |  | 1.07 (0.96-1.18)^#^ |
| 9.0-9.9 | 1,020 | 613 | 71.92 (66.23-77.62) |  | 1.10 (1.00-1.22)^\|\|^ |  | 1.42 (1.28-1.58)^¶^ |  | 1.23 (1.09-1.38)^#^ |
| ≥10.0 | 676 | 429 | 83.82 (75.89-91.73) |  | 1.58 (1.41-1.77)^\|\|^ |  | 1.84 (1.63-2.07)^¶^ |  | 1.32 (1.13-1.54)^#^ |

^*^ Inconsistency between total population and population summed for individual variable was due to missing information; HbA1c=glycated hemoglobin; CKD=chronic kidney disease; No anemia: Hb ≥ 13 in men and ≥12 g/dL in women; Anemia: Hb <13 in men and <12 g/dL in women.

^†^ Based on Poisson assumption, CI=confidence interval

^‡^ HR= hazard ratio; CI=confidence interval

^§^ p values for the interaction of mean HbA1c with mean hemoglobin and mean estimated glomerular filtration rate were < 0.0001 and 0.0007, respectively.

^||^ Based on Cox proportional hazard regression with adjustment for general characteristics (i.e., type of diabetes, age and sex)

^¶^ Based on Cox proportional hazard regression with adjustment for the general characteristics adjusted in Model 1 plus antidiabetic, antihypertensive, and antilipid medications.

^#^ Based on Cox proportional hazard regression with all covariates included in Model 2 plus comorbidities, complications, and laboratory results.
