# Supplementary material for: Low mean HbA1c does not increase all-cause and cardiovascular mortality in patients with diabetes: Effect-modifications by anemia and chronic kidney disease stages
Source: PLoS One. 2022 Aug 11;17(8):e0272137. doi: 10.1371/journal.pone.0272137 (PMC9371313; doi:10.1371/journal.pone.0272137)
Supplement: S4 Table — * Inconsistency between total population and population summed for individual variable was due to missing information; HbA1c = glycated hemoglobin; CKD = chronic kidney disease; No anemia: Hb ≥ 13 in men and ≥12 g/dL in women; Anemia: Hb <13 in men and <12 g/dL in women. † Based on Poisson assumption, CI = confidence interval. ‡ HR = hazard ratio; CI = confidence interval. § p values for the interaction of mean HbA1c with mean hemoglobin and mean estimated glomerular filtration rate were 0.2860 and 0.0022, respectively. || Based on Cox proportional hazard regression with adjustment for general characteristics (i.e., type of diabetes, age and sex). ¶ Based on Cox proportional hazard regression with adjustment for the general characteristics adjusted in Model 1 plus antidiabetic, antihypertensive, and antilipid medications. # Based on Cox proportional hazard regression with all covariates included in Model 2 plus comorbidities, complications, and laboratory result. (DOCX) [file pone.0272137.s004.docx]

S4 Table: Overall rates and relative hazards of cardiovascular mortality by different mean HbA1c levels with stratification by anemia and chronic kidney disease staging status

| Level of mean HbA1c (%)^*^ | Cardiovascular mortality | | |  | Model 1  Adjusted HR (95% CI)^‡^ |  | Model 2  Adjusted HR (95% CI)^‡^ |  | Model 3  Adjusted HR (95% CI)^‡,§^ |
| --- | --- | --- | --- | --- | --- | --- | --- | --- | --- |
|  | No. of patients | No. of mortality | Rates (per 1,000 patient-years)  (95% CI)^†^ |  |  |  |  |  |  |
| **No Anemia** |  |  |  |  |  |  |  |  |  |
| Stages 1-2 CKD |  |  |  |  |  |  |  |  |  |
| <6.0 | 442 | 12 | 4.84 (2.10-7.57) |  | 1.86 (1.00-3.45)^\|\|^ |  | 1.39 (0.74-2.59)^¶^ |  | 1.55 (0.78-3.10)^#^ |
| 6.0-6.9 | 5,064 | 67 | 2.26 (1.72-2.81) |  | 1.0 (Reference) |  | 1.0 (Reference) |  | 1.0 (Reference) |
| 7.0-7.9 | 5,593 | 96 | 2.44 (1.95-2.93) |  | 1.06 (0.78-1.45)^\|\|^ |  | 1.58 (1.13-2.19)^¶^ |  | 1.43 (0.98-2.09)^#^ |
| 8.0-8.9 | 2,858 | 70 | 3.39 (2.59-4.18) |  | 1.72 (1.23-2.41)^\|\|^ |  | 2.98 (2.05-4.33)^¶^ |  | 2.26 (1.44-3.56)^#^ |
| 9.0-9.9 | 1,354 | 37 | 3.85 (2.61-5.08) |  | 2.13 (1.42-3.20)^\|\|^ |  | 3.56 (2.27-5.60)^¶^ |  | 2.09 (1.15-3.79)^#^ |
| ≥10.0 | 1,099 | 40 | 5.27 (3.64-6.90) |  | 3.42 (2.29-5.12)^\|\|^ |  | 5.22 (3.33-8.19)^¶^ |  | 3.02 (1.56-5.84)^#^ |
| Stages 3-5 CKD |  |  |  |  |  |  |  |  |  |
| <6.0 | 183 | 12 | 9.47 (4.11-14.83) |  | 1.80 (0.98-3.32)^\|\|^ |  | 1.06 (0.56-1.98)^¶^ |  | 1.44 (0.75-2.74)^#^ |
| 6.0-6.9 | 1,854 | 71 | 5.36 (4.11-6.60) |  | 1.0 (Reference) |  | 1.0 (Reference) |  | 1.0 (Reference) |
| 7.0-7.9 | 2,339 | 99 | 4.81 (3.87-5.76) |  | 0.93 (0.68-1.27)^\|\|^ |  | 1.37 (1.00-1.90)^¶^ |  | 1.32 (0.92-1.90)^#^ |
| 8.0-8.9 | 1,316 | 71 | 5.84 (4.48-7.19) |  | 1.08 (0.78-1.51)^\|\|^ |  | 1.97 (1.35-2.86)^¶^ |  | 1.73 (1.12-2.67)^#^ |
| 9.0-9.9 | 619 | 30 | 5.19 (3.33-7.04) |  | 1.15 (0.74-1.77)^\|\|^ |  | 1.98 (1.23-3.20)^¶^ |  | 1.41 (0.80-2.48)^#^ |
| ≥10.0 | 359 | 38 | 11.97 (8.17-15.78) |  | 3.04 (2.03-4.55)^\|\|^ |  | 5.11 (3.25-8.02)^¶^ |  | 2.22 (1.12-4.40)^#^ |
| **Anemia** |  |  |  |  |  |  |  |  |  |
| Stages 1-2 CKD |  |  |  |  |  |  |  |  |  |
| <6.0 | 202 | 15 | 13.54 (6.69-20.39) |  | 1.16 (0.66-2.03)^\|\|^ |  | 1.13 (0.64-1.99)^¶^ |  | 1.05 (0.57-1.94)^#^ |
| 6.0-6.9 | 1,231 | 66 | 9.19 (6.98-11.41) |  | 1.0 (Reference) |  | 1.0 (Reference) |  | 1.0 (Reference) |
| 7.0-7.9 | 1,465 | 67 | 6.68 (5.08-8.29) |  | 0.68 (0.48-0.95)^\|\|^ |  | 0.91 (0.64-1.30)^¶^ |  | 0.89 (0.60-1.32)^#^ |
| 8.0-8.9 | 851 | 33 | 5.66 (3.73-7.59) |  | 0.66 (0.43-1.00)^\|\|^ |  | 1.09 (0.69-1.71)^¶^ |  | 1.06 (0.63-1.78)^#^ |
| 9.0-9.9 | 462 | 43 | 13.34 (9.35-17.33) |  | 1.73 (1.18-2.56)^\|\|^ |  | 2.83 (1.84-4.36)^¶^ |  | 2.46 (1.43-4.22)^#^ |
| ≥10.0 | 413 | 27 | 10.20 (6.36-14.05) |  | 1.77 (1.12-2.81)^\|\|^ |  | 2.99 (1.78-5.01)^¶^ |  | 2.71 (1.36-5.38)^#^ |
| Stages 3-5 CKD |  |  |  |  |  |  |  |  |  |
| <6.0 | 397 | 32 | 13.84 (9.04-18.63) |  | 0.97 (0.67-1.41)^\|\|^ |  | 0.80 (0.55-1.17)^¶^ |  | 0.68 (0.45-1.03)^#^ |
| 6.0-6.9 | 2,238 | 224 | 14.33 (12.45-16.21) |  | 1.0 (Reference) |  | 1.0 (Reference) |  | 1.0 (Reference) |
| 7.0-7.9 | 3,061 | 348 | 13.72 (12.28-15.17) |  | 0.93 (0.78-1.10)^\|\|^ |  | 1.20 (1.01-1.43)^¶^ |  | 1.20 (0.99-1.46)^#^ |
| 8.0-8.9 | 2,070 | 221 | 12.30 (10.68-13.92) |  | 0.93 (0.77-1.12)^\|\|^ |  | 1.29 (1.05-1.58)^¶^ |  | 1.20 (0.95-1.52)^#^ |
| 9.0-9.9 | 1,020 | 110 | 12.91 (10.49-15.32) |  | 1.07 (0.85-1.35)^\|\|^ |  | 1.49 (1.17-1.91)^¶^ |  | 1.36 (1.03-1.81)^#^ |
| ≥10.0 | 676 | 77 | 15.05 (11.68-18.41) |  | 1.55 (1.19-2.02)^\|\|^ |  | 2.05 (1.54-2.72)^¶^ |  | 1.39 (0.97-2.00)^#^ |

^*^ Inconsistency between total population and population summed for individual variable was due to missing information; HbA1c=glycated hemoglobin; CKD=chronic kidney disease; No anemia: Hb ≥ 13 in men and ≥12 g/dL in women; Anemia: Hb <13 in men and <12 g/dL in women.

^†^ Based on Poisson assumption, CI=confidence interval

^‡^ HR= hazard ratio; CI=confidence interval

^§^ p values for the interaction of mean HbA1c with mean hemoglobin and mean estimated glomerular filtration rate were 0.2860 and 0.0022, respectively.

^||^ Based on Cox proportional hazard regression with adjustment for general characteristics (i.e., type of diabetes, age and sex)

^¶^ Based on Cox proportional hazard regression with adjustment for the general characteristics adjusted in Model 1 plus antidiabetic, antihypertensive, and antilipid medications.

^#^ Based on Cox proportional hazard regression with all covariates included in Model 2 plus comorbidities, complications, and laboratory results.
